# Identification and Validation of *qSTS5*, a QTL Associated with Salt Tolerance at Seedling Stage in Dongxiang Wild Rice

**DOI:** 10.3390/biology15090702

**Published:** 2026-04-29

**Authors:** Yi-Jie Yan, Yu-Jun Zhu, Zhen-Hua Zhang, Ling Wang, Feng-Li Zhao, Yu-Song Lyu, Gao-Neng Shao, Li-Hong Xie, Gui-Ai Jiao, Shi-Kai Hu, Zhong-Hua Sheng, Biao-Lin Hu, Bo Shen, Shao-Qing Tang

**Affiliations:** 1 State Key Laboratory of Rice Biology and Breeding, China National Rice Research Institute, Hangzhou 310006, Chinazhuyujun@caas.cn (Y.-J.Z.); zhangzhenhua@caas.cn (Z.-H.Z.); wangling03@caas.cn (L.W.); zhaofengli@caas.cn (F.-L.Z.); lvyusong@caas.cn (Y.-S.L.); shaogaoneng@caas.cn (G.-N.S.); xielihong@caas.cn (L.-H.X.); jiaoguiai@caas.cn (G.-A.J.); hushikai@caas.cn (S.-K.H.); shengzhonghua@caas.cn (Z.-H.S.); 2 College of Life and Environmental Sciences, Hangzhou Normal University, Hangzhou 311121, China; 3 Jiangxi Early-Season Rice Research Centre, China National Rice Research Institute, Pingxiang 337029, China; 4 Rice Research Institute, Jiangxi Academy of Agricultural Sciences, Nanchang 330200, China; hubiaolin992@126.com

**Keywords:** Dongxiang wild rice, seedling stage, salt tolerance, QTL mapping, transcriptome sequencing

## Abstract

Soil salinization severely restricts rice growth and global grain production, posing a serious threat to food security. Dongxiang wild rice serves as an important genetic resource for improving salt tolerance in rice. In this study, a backcross inbred line (BIL) population derived from Dongxiang wild rice DY80 and an *indica* restorer line R974 were used to detect QTLs for salt tolerance at the germination and seedling stages. Four QTLs related to germination-stage salt tolerance and three QTLs for seedling-stage salt tolerance were identified, among which *qSTS5* on chromosome 5 showed the largest effect with a *LOD* score of 8.0 and a phenotypic contribution rate of 14.8%. An F_2:3_ population was further constructed to validate *qSTS5*, which increased its *LOD* value to 10.4 and phenotypic variation explanation rate to 18.5%, and the locus was finally delimited to a 2.3 Mb interval. Transcriptome analysis identified eight differentially expressed genes (DEGs) within the *qSTS5* region under salt stress. Sequence comparison between the parents revealed that three DEGs had no coding-region variations, while the other five showed nucleotide polymorphisms leading to amino acid changes. Among them, *Os05g0349800* encodes a LEA protein, a typical stress-responsive gene, and harbors a frameshift mutation in DY80. Combined with its induced expression pattern under salt stress, this gene was considered the most promising candidate for *qSTS5*. This study not only provides a stable major QTL for rice breeding for salt tolerance but also lays a foundation for dissecting the molecular mechanism of salt tolerance in Dongxiang wild rice.

## Simple Summary

Soil salinization limits rice growth and threatens food security. Using a Dongxiang wild rice-derived BIL population, we identified four germination-stage and three seedling-stage salt-tolerance QTLs, with the major locus *qSTS5* showing the strongest effect. QTL validation delimited it to a 2.3 Mb region. Transcriptome and sequence analyses highlighted the LEA gene *Os05g0349800* as the key candidate. This study provides a valuable locus for salt-tolerant rice breeding.

## 1. Introduction

Soil salinization is one of the major abiotic stresses contributing to soil degradation and constraining global food crop production, significantly impairing plant growth and development [1]. Under the dual pressures of intensifying climate change and improper irrigation practices, salinization is progressively encroaching upon regions previously unaffected, leading to a continuous expansion of salt-affected land area worldwide [2]. It is estimated that approximately 40% of the world’s irrigated cropland has been adversely impacted by soil salt accumulation [3,4]. China is among the countries most severely affected by soil salinization, and the extensive presence of saline–alkali land substantially limits agricultural productivity and poses a serious threat to national food security [5]. Therefore, the scientific utilization and sustainable management of salt-affected lands hold strategic significance for expanding arable land area and enhancing grain production capacity in China.

Rice (*Oryza sativa* L.) is one of the most important staple crops in the world, serving as the primary dietary source for approximately 3.5 billion people globally, and it plays an irreplaceable role in ensuring food security, particularly across Asia [6,7]. However, rice is highly sensitive to salt stress, especially during the seedling and reproductive stages. In this context, the identification of key salt-tolerance genes and the accelerated development of salt-tolerant rice varieties have become urgent priorities and critical strategies for safeguarding food security both in China and globally.

The response of rice to salt stress is a highly complex biological process, primarily involving the regulation of ion homeostasis, osmotic adjustment, reactive oxygen species (ROS) scavenging, and phytohormone-mediated signaling pathways [8]. Among these mechanisms, the maintenance of Na^+^/K^+^ homeostasis is a critical determinant of salt tolerance in rice [8]. Under salt stress, Na^+^, as the main toxic ion, is excessively taken up by plant roots. To mitigate Na^+^-induced toxicity, plants employ multiple strategies, including suppression of Na^+^ uptake, enhancement of Na^+^ efflux, and compartmentalization of excess Na^+^ into the vacuole, thereby effectively alleviating ionic stress and improving salt tolerance [8,9,10]. In parallel, plants synthesize and accumulate compatible solutes, such as proline, glycine betaine, and trehalose, to maintain cellular osmotic balance [9,11]. Furthermore, a network of phytohormones, including abscisic acid (ABA), gibberellins (GA), cytokinins (CTK), ethylene and so on, orchestrates stress responses through intricate cross-talk and regulatory interactions [8,12,13,14]. Concurrently, the activation of antioxidant defense systems such as Superoxide dismutase (SOD) and Catalase (CAT) enables efficient scavenging of ROS generated under salt stress [11]. Collectively, these interconnected physiological and molecular pathways constitute an integrated defense system for rice to cope with salt stress and enhance its overall salt tolerance.

Salt tolerance in rice is a classic quantitative trait, controlled by multiple genes and significantly influenced by genotype–environment interactions [15]. Conventional single-gene functional studies are insufficient to fully elucidate its complex genetic architecture. Therefore, QTL mapping based on segregating populations has emerged as an effective strategy for identifying major-effect loci associated with salt tolerance. To date, numerous QTLs related to salt tolerance have been reported, but only a limited number exhibiting substantial phenotypic effects have been successfully isolated or fine-mapped [15,16]. The majority of identified salt-tolerance QTLs are associated with traits at the germination, seedling, and vegetative–reproductive stages and are distributed on the twelve chromosomes of rice, with the most QTLs distributed on chromosome 1. These QTLs have been linked to various physiological traits, including germination rate, survival rate, seedling height, root length, and Na^+^/K^+^ content [17,18]. Using an F_2:3_ population constructed by the highly salt-tolerant *indica* variety Nona Bokra and the salt-sensitive elite japonica cultivar Koshihikari, QTL analysis based on physiological salt-tolerance traits identified several significant loci. Among them, two major QTLs, *qSNC-7* (*LOD* = 7.66), associated with shoot Na^+^ concentration, and *qSKC-1* (*LOD* = 11.74), associated with shoot K^+^ concentration, explained 48.5% and 40.1% of the total phenotypic variance, respectively [18]. Subsequently, Ren et al. [19] employed a map-based cloning strategy to fine-map *qSKC-1* to a 7.4 kb genomic interval and successfully isolated the underlying causal gene, designated *SKC1*. *SKC1* encodes OsHKT1;5, an ion transporter belonging to the *HKT* family. This protein is preferentially expressed in xylem parenchyma cells surrounding the vascular bundles with the function of transporting Na^+^, thereby maintaining the Na^+^/K^+^ homeostasis in rice and enhancing the ability of rice to resist salt stress [19,20]. In addition, a QTL significantly associated with shoot Na^+^/K^+^ ratio at the seedling stage, designated *Saltol*, was identified from the salt-tolerant landrace Pokkali. In the RIL population, *Saltol* explained 43.2% of the phenotypic variation in the Na^+^/K^+^ ratio [21]. Given the close physical proximity of *Saltol* and *qSKC-1* on the chromosome and their shared role in regulating Na^+^/K^+^ homeostasis, it has been proposed that both loci likely correspond to the same functional gene, *OsHKT1;5* [22].

Wild rice (*Oryza rufipogon* Griff.) is the ancestor of cultivated rice (*Oryza sativa* L.). During prolonged artificial domestication and selection, cultivated rice has experienced a substantial reduction in genetic diversity, resulting in a markedly narrowed gene pool [23]. In contrast, wild rice, shaped by long-term natural selection, has evolved extensive genetic variation and robust environmental adaptability. It retains numerous favorable alleles that were lost or depleted during the domestication of cultivated rice and has strong tolerance in resistance to biotic stresses such as rice blast and bacterial blight and abiotic stresses such as drought and cold resistance [24,25], making it an invaluable reservoir of genetic resources for rice improvement. Dongxiang wild rice, a representative wild rice germplasm native to Jiangxi Province, China, not only possesses notable cold and submergence tolerance but also displays significantly enhanced salt tolerance compared to cultivated rice [26]. So far, multiple QTLs related to biotic and abiotic stress resistance have been successfully identified from Dongxiang wild rice [27,28,29]. Functional validation further demonstrates that introgression of genomic segments from Dongxiang wild rice into cultivated backgrounds can substantially enhance salt tolerance in elite rice varieties [30]. Therefore, using Dongxiang wild rice as a donor parent in interspecific crosses, combined with the QTL mapping strategy, offers a promising avenue for isolating novel, major-effect salt-tolerance genes absent in modern cultivars. This approach not only holds potential to overcome current bottlenecks in salt-tolerance breeding but also provides critical genetic resources for developing high-yielding, stress-resilient rice varieties, thereby contributing strategically to sustainable rice production in the face of escalating soil salinization [31].

In this study, a backcross inbred line (BIL) population derived from Dongxiang wild rice DY80 and an *indica* restorer line R974 was used to detect QTLs for salt tolerance at the germination and seedling stages. One major-effect QTL associated with salt tolerance at the seedling stage, *qSTS5*, was identified. By integrating QTL mapping and RNA-seq, eight DEGs were identified in the *qSTS5* interval, and the parental sequences were analyzed. Among them, *Os05g0349800* encodes a LEA protein, a typical stress-responsive gene, and harbors a frameshift mutation in DY80. Combined with its induced expression pattern under salt stress, this gene was considered the most promising candidate for *qSTS5*. This study not only provides a stable major QTL for rice breeding for salt tolerance but also lays a foundation for dissecting the molecular mechanism of salt tolerance in Dongxiang wild rice.

## 2. Materials and Methods

### 2.1. Rice Materials

**Figure 1:**
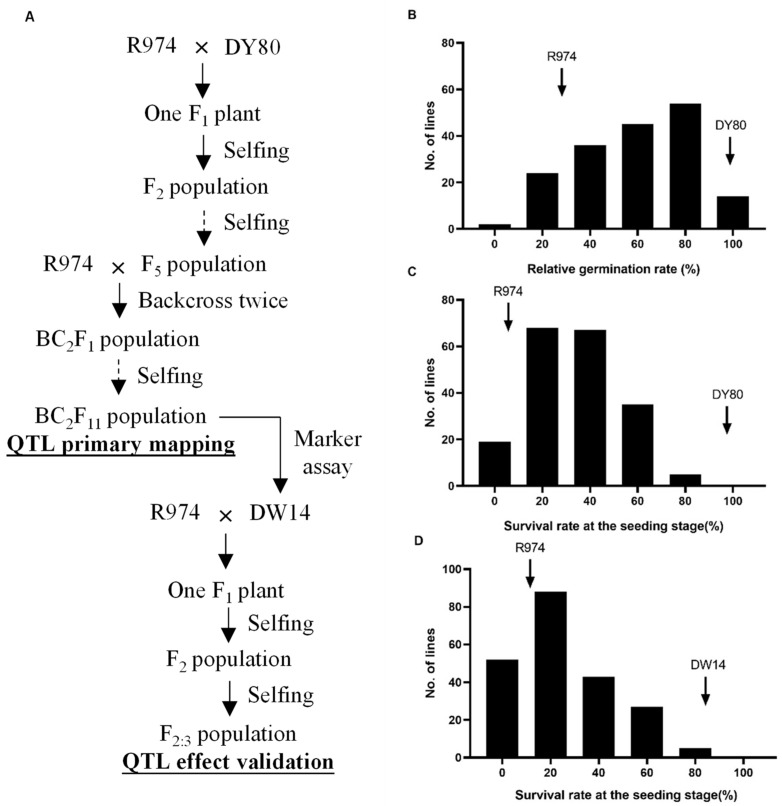
Construction of the genetic populations and phenotypic evaluation. (**A**) Development of the primary mapping population and the secondary validation population. (**B**,**C**) Phenotypic assessment of salt tolerance in the primary mapping population at the germination and seedling stages, respectively. (**D**) Phenotypic assessment of salt tolerance in the secondary validation population at the seedling stage.

Two mapping populations were used in this study. One was a BIL population, consisting of 206 lines, used for the primary mapping of QTL associated with salt tolerance at the germination and seedling stages. This population was derived from a cross between DY80 and R974. DY80 was a common wild rice line found growing in Dongxiang County, Jiangxi Province, China. R974 was a representative *indica* restorer variety (Figure 1A). The process of population construction is described as follows. The initial cross was generated using R974 as the female parent and DY80 as the male parent. A resulting F_1_ plant was self-pollinated to produce an F_2_ population. Each F_2_ single plant was subsequently selfed for 3 consecutive generations to advance an F_5_ population. Then, R974 was used as the recurrent parent in 2 successive backcrosses with each F_5_ line, generating a BC_2_F_1_ population consisting of 206 lines. Each BC_2_F_1_ line was selfed for 10 consecutive generations to generate the BC_2_F_11_ population, comprising 206 lines (Figure 1A).

The other one was an F_2:3_ population, which was utilized to validate *qSTS5*, showing a significant genetic effect on salt tolerance at the seedling stage as identified in the primary mapping. The process of this population construction was described as follows. A new cross was established between the recurrent parent R974 and DW14, a highly salt-tolerant line at the seedling stage from the BILs. The resulting F_1_ plant was selfed to produce an F_2_ population. Individual F_2_ plants were harvested separately and advanced to produce corresponding F_3_ family lines, generating an F_2:3_ population comprising 215 lines (Figure 1A). The seeds of each line were utilized for salt-tolerance identification at the seedling stage following harvest.

### 2.2. Salt-Tolerance Evaluation at the Germination Stage

Thirty seeds from each BIL were placed in a Petri dish lined with filter paper. The control and treatment groups were set up, with three replicates for each treatment. The control and treatment groups were treated with 15 mL of deionized water (dd H_2_O) and 150 mmol/L NaCl, respectively. Both dishes were covered and placed in a constant-temperature incubator at 30 °C. Germination was observed daily and the germination rate was counted on the 10th day.

Germination Rate = (Number of Germinated Seeds/Total Number of Seeds) × 100%

Relative Germination Rate = (Treatment Group Germination Rate/Control Group Germination Rate) × 100%

### 2.3. Salt-Tolerance Evaluation at the Seedling Stage

Thirty seeds were selected from each line, which were soaked in water for 2 days and then germinated for another 2 days. Sixteen seeds with consistent germination were selected from each line and transferred to 96-well plates. Six lines were planted on each plate. They were first cultured in distilled water for 4 days, and then transferred to Yoshida nutrient solution for further growth. When the rice seedlings reached the three-leaf stage, they were transferred to a nutrient solution containing 150 mmol/L NaCl for 9 days. After that, the salt solution was replaced with a standard nutrient solution to carry out the recovery experiment for 5 days. The number of live and dead seedlings was recorded, and the survival rate was calculated. Three replicates were set up in the experiment. The seedlings were grown in a growth chamber under controlled environmental conditions of 28/23 °C day/night temperature and a 14 h light/10 h dark photoperiod. The nutrient solution or salt solution was refreshed every 3 days.

### 2.4. DNA Extraction and Genotype Detection

DNA extraction was performed using the simple method described by Zheng et al. [32]. PCR amplification was carried out according to the method of Chen et al. [33], using 2 × Taq Master Mix (Dye) (CWBIO, Taizhou, China). The PCR products were separated on 6% non-denaturing polyacrylamide gel and stained with silver nitrate.

### 2.5. Map Construction and QTL Analysis

The genetic linkage map was constructed using the “MAP (Linkage map construction in biparental populations)” function of the QTL IciMapping software (Version 4.2), with a LOD threshold of 3.0. QTL analysis utilized the “BIP (QTL mapping in biparental populations)” module of the same software, employing the “ICIM-ADD” mapping method. The LOD threshold for declaring significant QTLs was determined based on 1000 permutations (*p* = 0.05). QTLs were named following the rules proposed by McCouch et al. [34].

### 2.6. Transcriptome Analysis

The transcriptome sequencing workflow includes RNA extraction, RNA detection, library construction, and on-machine sequencing. After 3 h, 12 h, 24 h and 48 h of saline stress treatment, the whole plant samples from the control and treatment groups were collected, immediately frozen in liquid nitrogen, and stored at −80 °C, with three in dependent biological replicates prepared for each sample. Total RNA was extracted from the whole plants using the RNeasy Plus Mini Kit (Qiagen, Hilden, Germany), and high-quality RNA samples were selected for high-throughput sequencing. Transcriptome sequencing was completed by Novogene Technology Co., Ltd. (Beijing, China). The Nipponbare (*Oryza sativa* L. ssp. *japonica* cv. Nipponbare) reference genome was downloaded from the National Center for Biotechnology Information (NCBI), and clean reads were aligned to the reference genome using HISAT2 (version 2.2.1). FPKM (fragments Per Kilobase of transcript Per Million fragments mapped) was used as a measure of transcript or gene expression levels.

### 2.7. qRT-PCR Validation of the DEGs

Gene-specific primers for qRT-PCR were designed from the cDNA sequences of the DEGs. First-strand cDNA was synthesized from 1 μg of total RNA using the ReverTra Ace^®^ qPCR RT Master Mix (Toyobo Co., Ltd., Osaka, Japan) according to the manufacturer’s instructions. qRT-PCR was performed on the CFX96TM fluorescence quantitative PCR instrument (Bio-Rad, Hercules, CA, USA) using SYBR Green-based detection. The rice *Actin* gene (*Os03g0718100*) was utilized as an internal standard. Each sample was analyzed with three biological replicates, and the 2^−ΔΔCT^ method was used to calculate the relative expression levels of genes. The sequence information for each primer is listed in Table S1.

### 2.8. Sequence Analysis

The DEGs located in the *qSTS5* region were subjected to sequence alignment analysis between the two parents, R974 and DY80. Genomic DNA was extracted from fresh leaves of the two parental lines. The PCR products amplified using primers listed in Table S2 were sequenced by Sanger sequencing. The nucleotide and predicted amino acid sequences of R974 and DY80 were compared.

## 3. Results

### 3.1. Salt-Tolerance Evaluation at the Germination and Seedling Stages in the BIL Population

Prior to the salt-tolerance evaluation of the BILs at the germination and seedling stages, R974 and DY80 were treated with four NaCl concentrations (50 mmol/L, 100 mmol/L, 150 mmol/L and 200 mmol/L). At the germination stage, no significant difference in relative germination rate was observed between the two parental lines at the 50 mmol/L and 200 mmol/L treatments. At 50 mmol/L, both parents exhibited a relative germination rate of 100.0%. At 200 mmol/L, the relative germination rates for both were below 5.0%. However, obvious differences were observed at 100 mmol/L and 150 mmol/L. The relative germination rate was recorded specifically at 150 mmol/L, revealing 36.7% for R974 and 99.2% for DY80, respectively. At the seedling stage, no significant difference was also observed between the two parental lines under the 50 mmol/L and 200 mmol/L saline treatments, with survival rates exceeding 90.0% and falling below 5.0%, respectively. However, obvious differences were observed at 100 mmol/L and 150 mmol/L. Survival rates were also recorded specifically at 150 mmol/L, with 15.3% for R974 and 90.1% for DY80. Based on the above results, a NaCl concentration of 150 mmol/L was selected for evaluating salt tolerance at both the germination and seedling stages.

Descriptive statistics for salt tolerance at the germination stage (STG) and salt tolerance at the seedling stage (STS) in the BIL population are presented in Table 1. The STG ranged from 0.0% to 98.3%, with skewness and kurtosis values of −0.3 and −1.0, respectively. The STS ranged from 0.0% to 82.5%, with skewness and kurtosis values of 0.4 and −0.3, respectively. Both traits exhibit a continuous distribution pattern with low skewness and kurtosis, showing a typical variation pattern of quantitative traits (Table 1, Figure 1B,C). Furthermore, for both traits, the trait values of DY80 are significantly higher than those of R974, suggesting that DY80 may possess genetic factors associated with salt tolerance.

### 3.2. QTL Primary Mapping for STG and STS

**Figure 2:**
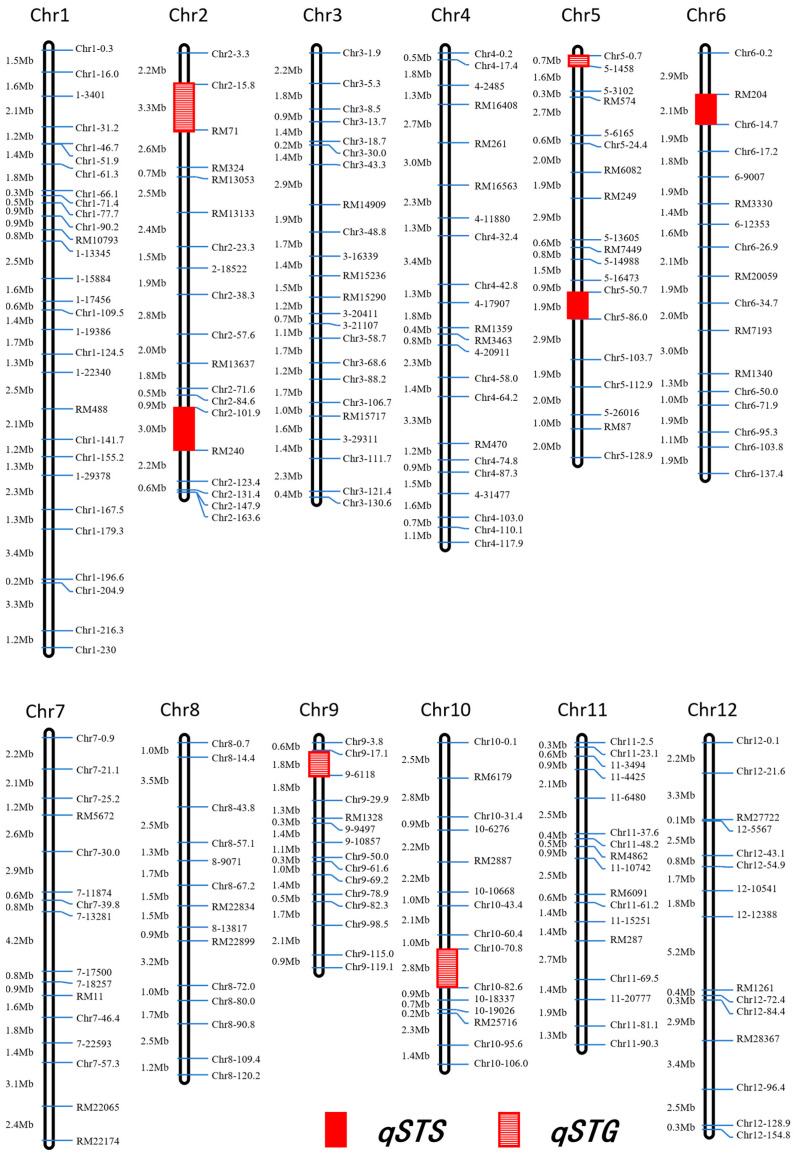
Chromosomal location of QTLs for the two traits. Markers distributed on each linkage group are shown on the right side of the chromosome, with physical distances (Mb) displayed on the left. Hollow red boxes denote QTLs associated with STG; solid red boxes denote QTLs associated with STS. STG, salt tolerance at the germination stage; STS, salt tolerance at the seedling stage.

The genetic map of BILs comprises 221 molecular markers, consisting of 44 SSRs, 49 KASPs, and 128 InDels (Table S3). The map spans 1356.7 cM, with an average inter-marker distance of 6.5 cM. The maximum and minimum genetic distances between adjacent markers are 20.8 cM and 0.1 cM, respectively (Figure 2).

The results of QTL analysis for STG and STS are presented in Table 2. For STG, four QTLs were detected, distributed on chromosomes 2, 5, 9, and 10, respectively (Figure 2). Except for *qSTG9*, the salt-tolerance alleles of the other three QTLs were all from DY80. *qSTG9* has the largest genetic effect, with a *LOD* score of 4.0 and an *R*^2^ of 8.6%. The allele from R974 increased the germination rate by 9.4%. The *LOD* scores of the other three QTLs were 2.9, 3.4, and 2.7, and the *R*^2^ values were 7.5%, 6.3%, and 5.1%, respectively. The allele from DY80 increased the germination rate by 9.7%, 8.7%, and 11.5% respectively (Table 2).

For STS, three QTLs were detected, which were distributed on chromosomes 2, 5, and 6, respectively (Figure 2). The salt-tolerance alleles of these three QTLs were all from DY80, among which *qSTS5* had the largest genetic effect, with a *LOD* score of 8.0 and an *R*^2^ of 14.8%, and the allele from DY80 improved the survival rate by 9.8%. The *LOD* scores of the other two QTLs were 2.7 and 2.8, respectively, and the *R*^2^ values were 3.9% and 7.2%, respectively. The allele from DY80 improved the survival rate by 3.9% and 7.2%, respectively. Comparative analysis of the *LOD* scores and *R*^2^ values of the QTLs associated with STG and STS revealed that *qSTS5* exhibited the highest *LOD* score and largest *R*^2^ (Table 2). Consequently, *qSTS5* was selected as the candidate locus for subsequent genetic validation. In addition, because no significant genetic effect on STG was detected in the primary mapping of *qSTS5* region, we only determined STS in the secondary mapping.

### 3.3. Genetic Effect Validation of qSTS5

Given the significant genetic effect of *qSTS5* on STS in the primary mapping, a secondary population was developed to validate the genetic effect of this region. A highly salt-tolerant line, DW14, carrying seven DY80 chromosomal fragments—RM488–CHR1-179.3 on chromosome 1, CHR2-84.6 on chromosome 2, CHR5-0.7–RM7449 and CHR5-50.7–CHR5-86.0 on chromosome 5, CHR10-106.0 on chromosome 10, CHR11-69.5–CHR11-90.3 on chromosome 11, and CHR12-43.1–CHR12-84.4 on chromosome 12 (Figure S1)—was selected from the BILs as the male parent and backcrossed with the recurrent parent R974 to generate an F_2:3_ population consisting of 215 lines. Phenotypic evaluation of STS showed that the survival rate across the population ranged from 0.0% to 87.5%, with skewness and kurtosis values of -0.1 and 0.8, respectively, showing a typical variation pattern of quantitative traits (Figure 1D). Two QTLs, *qSTS5* and *qSTS12*, were detected. The genetic effect of *qSTS5* was obviously greater than that of *qSTS12*, with an *R*^2^ of 18.5%. The DY80 allele increased the survival rate by 12.0%. In comparison, *qSTS12* explained 5.2% of the phenotypic variation, and the DY80 allele increased survival rate by 7.0% (Table 3).

These results suggested that *qSTS5* had a significant genetic effect on salt tolerance at the seedling stage. Compared with the primary mapping, the genetic effect of *qSTS5* was further enhanced in the secondary population, with the *LOD* score increasing from 8.0 to 10.4, the *R*^2^ rising from 14.8% to 18.5%, and the additive effect increasing from 9.8% to 12.0%. To further narrow the region of *qSTS5*, polymorphic markers were designed and added in the crossover region between CHR5-50.7 and 5-16473 and between CHR5-86.0 and CHR5-103.7, respectively, based on the sequence variation between R974 and DY80 (Figure S1). Two KASP markers, 5-17249 and 5-19507, were added to flank CHR5-50.7 and CHR5-86.0, respectively. Marker analysis revealed that both loci (5-17249 and 5-19507) are R974 homozygous (Figure S1). Therefore, *qSTS5* was delimited by the markers 5-17249 and 5-19507, corresponding to a 2.3 Mb region of Nipponbare genome.

### 3.4. Transcriptome Analysis of the Highly Salt-Tolerant Line

**Figure 3:**
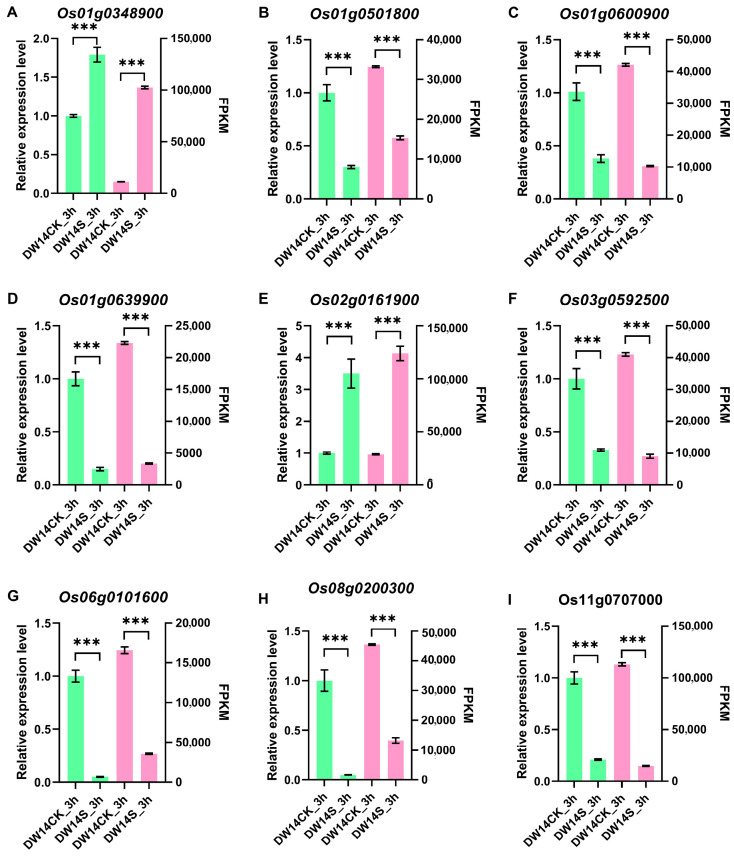
qPCR analysis of the control (DW14CK_3h) and saline-treated (DW14S_3h) groups of DW14 after a 3 h saline treatment. The green and pink bar charts showed the qPCR and FPKM values, respectively. (**A**–**I**) qPCR and FPKM values of differential genes. Values are given as the mean ± SD (*n* = 3). Significant difference was detected by using Student’s *t*-test. *** *p* < 0.001.

Given the large and stable genetic effect of *qSTS5*, we aim to identify candidate genes potentially regulating salt tolerance at the seedling stage in this 2.3 Mb region. Previous studies have shown that most rice stress tolerance traits are associated with the expression level of genes. Therefore, mRNA-seq analysis was conducted to identify candidate genes likely involved in the regulation of *qSTS5*. DW14, a highly salt-tolerant line, was used for saline treatment, and the whole plants of the control group and the treatment group were sampled at 3 h, 12 h, 24 h and 48 h after saline treatment. Following transcriptome data analysis, nine differentialy expressed genes (DEGs) identified at 3 h were randomly selected for validation via qPCR. The results demonstrated that the qPCR expression trends were consistent with those obtained from transcriptome sequencing (Figure 3).

**Figure 4:**
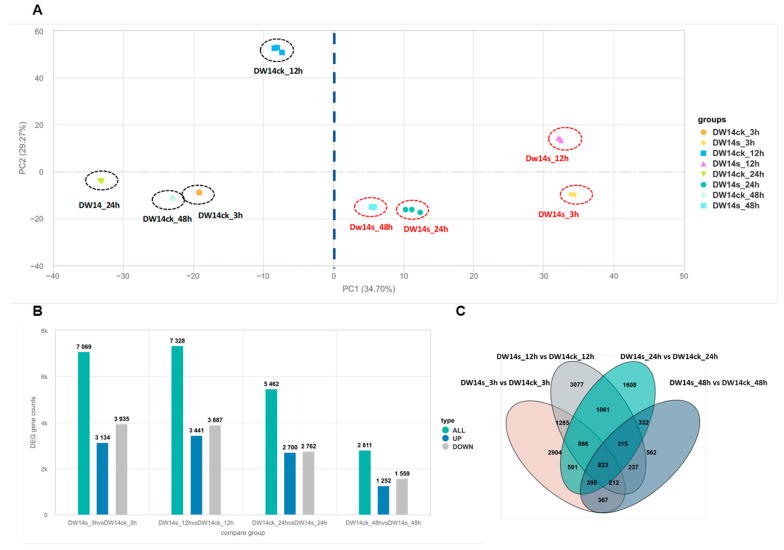
Transcriptomic changes in DW14 under saline stress following 3 h, 12 h, 24 h, and 48 h treatments. (**A**) Principal component analysis (PCA) of all gene transcripts of DW14 before and after saline treatment. PC1 and PC2 represented the first and second principal components, respectively. (**B**) Statistics of up-regulated and down-regulated differentially expressed genes (DEGs) of DW14 before and after saline treatment. (**C**) Wayne diagram of differentially expressed genes among different times.

A total of 172.1 Gb of data was obtained and the quality was verified (Table S4). A total of 35,254 expressed genes were detected, including 31,761 known genes and 3493 new genes. Then, principal component analysis (PCA) was used to visualize the transcriptional variation in these samples. Under stress conditions, the PCA map clearly distinguished two groups. Taking PC1 as a representative, 34.70% of the total variation was explained, indicating that the transcription level changed greatly with the saline treatment (Figure 4A).

Compared with the control group, 7069 (3134 up-regulated, 3935 down-regulated), 7328 (3441 up-regulated, 3887 down-regulated), 5462 (2700 up-regulated, 2762 down-regulated), and 2811 (1252 up-regulated, 1559 down-regulated) DEGs were identified after 3, 12, 24, and 48 h of saline treatment, using a threshold of log2|fold change| > 1.0 (Figure 4B).

Among these genes, 2904, 3077, 1608, and 562 DEGs were uniquely identified at 3 h, 12 h, 24 h, and 48 h, respectively. A total of 823 DEGs were shared across all sampling times (Figure 4C), and eight of them were located in the *qSTS5* region. The expression levels of five genes were significantly up-regulated after saline treatment, while the remaining three genes were significantly down-regulated (Table S5). In the following experiments, these eight DEGs were further analyzed to identify the candidate gene underlying *qSTS5*.

### 3.5. Sequence Analysis of the Eight DEGs

According to the Rice Annotation Project Database, five DEGs encode proteins with known functional domains. *Os05g0349800* encodes an embryonic abundant protein. *Os05g0361700* encodes an AP2 domain-containing protein. *Os05g0373900* encodes a eukaryotic peptide chain release factor subunit 1-1. *Os05g0381400* encodes a plasma membrane protein 1, OsPM1. *Os05g0399400* encodes a chitinase family protein precursor, Cht9. The remaining three encode unknown functional domains. *Os05g0331900* and *Os05g0390300* encode expressed proteins. *Os05g0341450* encodes a conserved hypothetical protein.

Sequence analyses of the eight genes between DY80 and R974 were performed. No mutation was detected in the coding regions of *Os05g0331900*, *Os05g0341450*, and *Os05g0381400*. For *Os05g0349800*, one SNP (G140T) and one 1 bp deletion (G141-) were identified in the coding region, resulting in a frameshift mutation at the 48th amino acid. For *Os05g0361700*, one SNP (A50G) was identified, leading to an amino acid substitution (H17R). For *Os05g0373900*, *Os05g0390300*, and *Os05g0399400*, 28, six and 12 SNPs were identified, resulting in four, three, and two amino acid substitutions, respectively (Table 4).

## 4. Discussion

### 4.1. Correlation Analysis of QTLs for Salt Tolerance at the Germination and Seedling Stages

Rice exhibits stage-specific sensitivity to salinity stress, with germination and seedling establishment representing two critical early developmental phases. Salt tolerance at these two stages profoundly influences seedling survival and subsequent growth performance under saline conditions. In this study, QTL mapping conducted in a BIL population identified four QTLs associated with STG and three QTLs associated with STS. Notably, no genomic intervals overlapped between the STG- and STS-associated QTLs, suggesting distinct genetic architectures underlying salt tolerance at these two developmental stages within the genetic population in this study. Previous studies have also shown that the genomic intervals harboring salt-tolerance-associated QTLs identified at different growth stages rarely overlap. Yuan et al. [35] identified three QTLs for salt tolerance at germination and six at the seedling stage using a RIL population from the *indica* variety Luhui 99 and the *japonica* variety Shennong 265, with no overlapping QTL regions. Nakhla et al. [36] detected salt-tolerance QTLs at multiple growth stages in a BIL population derived from the African rice ACC9 and the *indica* variety Zhenshan 97; most QTLs showed stage specificity, though some intervals overlapped across stages. Chapagain et al. [37] mapped salt-tolerance QTLs at germination, seedling and reproductive stages using an introgression line population from the U.S. rice Cheniere and the tropical *japonica* variety TCCP. Only *qSG5.8* and *qMGT6.12* (germination) overlapped with *qSRR5.8* and *qMGT6.12* (seedling), while most other QTLs were stage-specific. These results imply that salt tolerance at germination and seedling stages may be controlled by different molecular mechanisms, requiring further research.

### 4.2. qSTS5 May Have Significant Salt Tolerance in Different Genetic Backgrounds

Mining stably expressed, large-effect salt-tolerance QTLs across various genetic backgrounds provides important genetic resources for salt-tolerant rice breeding. Based on the physical location of *qSTS5*, it was found that the salt tolerance in this interval was detected in many different genetic populations. Three QTLs, *qSES5* [38], *qDSRs5-1* [39] and *qST5-2* [40], were associated with seedling-stage salt tolerance and co-localize with *qSTS5*. *qSES5* was identified in a mapping population derived from Pokkali and IR29, with the salt-tolerance allele originating from Pokkali [38]. *qDSRs5-1* was identified in a population derived from Yiai1 and Lishuinuo, with the beneficial allele originating from Yiai1 [39]. *qST5-2* was identified in a population derived from Hunan Chaling common wild rice and *indica* rice 9311, with the beneficial allele originating from common wild rice [40]. *qSIS5.2* and *qRTL5.2* were QTLs associated with root length traits under salt stress, detected in a population derived from rice varieties Nona Bokra and Cheniere; both co-localize with *qSTS5* [41]. Ghomi K et al. identified eight QTLs associated with seedling-stage salt-tolerance traits on chromosome 5 using an F_2:4_ segregation population derived from Gharib and Sepidroud; among these, *qSFW-5a*, *qSDW-5a*, and *qBM-5a* co-localize with *qSTS5* [42]. Additionally, *qNaK5.1*, a QTL for the Na^+^/K^+^ ratio identified from a Hasawi × BRRI dhan28 population, also co-localizes with *qSTS5* [43]. A salt-tolerance QTL with the same name detected via GWAS also overlaps the physical position of *qSTS5* [44]. Independent studies using diverse germplasms consistently identified salt-tolerance QTLs in this region of chromosome 5, demonstrating that *qSTS5* provides stable salt tolerance with little genetic background effect.

### 4.3. Genetic Background Homogeneity Can Improve the Efficiency of QTL Detection

The detection of QTLs is highly sensitive to confounding influences from genetic background, major-effect genes, and other experimental or biological factors, frequently resulting in failure to detect QTLs with small effects. *SG3* is a QTL that regulates rice grain length [45]. During primary mapping, *SG3* exhibited only a minor effect on grain length due to the influence of the major-effect grain length QTL *GS3*. When the genetic effect of *GS3* was eliminated, the effect of *SG3* on grain length increased significantly and was successfully cloned [45]. In this study, *qSTS12*, a QTL associated with STS, was mapped in the secondary population, although it had not been detected in the primary mapping. The detection of *qSTS12* likely benefited from the genetic background homogenization achieved in the secondary population. Compared with the primary population, which has a complex genetic background, the secondary population was only separated in a few intervals (Figure S1), thereby effectively minimizing genetic background interference and significantly enhancing QTL detection efficiency. In addition, genetic background homogenization facilitates more accurate evaluation of QTL genetic effects. In this study, the genetic effect and *R*^2^ of *qSTS5* were significantly enhanced in the secondary population: the *LOD* score increased from 8.0 to 10.4, the additive effect from 9.8% to 12.0%, and the *R*^2^ from 14.8% to 18.5%.

### 4.4. Analysis of the Candidate Genes in qSTS5 Region

RNA-seq, with high throughput, resolution and sensitivity, allows efficient identification of differentially expressed genes, but their large number and diverse functions hinder target gene screening. QTL mapping locates trait-related regions but often yields large intervals with many genes, and fine mapping is time-consuming. Combining QTL mapping with RNA-seq can overcome their respective limitations and accelerate the mining of target genes. In this study, by integrating QTL mapping and RNA-seq analyses, eight DEGs were identified within the *qSTS5* interval, and their parental sequences were compared. Three of these genes—*Os05g0331900*, *Os05g0341450*, and *Os05g0381400*—exhibited no sequence variations in the coding regions between the two parents. Four other genes, *Os05g0361700*, *Os05g0373900*, *Os05g0390300*, and *Os05g0399400*, displayed one, four, three, and two amino acid substitutions, respectively, yet their biological functions remained uncharacterized. The remaining gene, *Os05g0349800*, encodes a LEA protein, a well-known stress-responsive gene whose expression is up-regulated under salt, drought, and low-temperature stresses [46]. Its expression pattern across multiple time points under salt stress in this study was highly consistent with previous reports, further supporting its potential role in salt stress responses. Meanwhile, sequence analysis revealed a 1 bp deletion (G141-) in the coding region of DY80, leading to an amino acid frameshift and premature translation termination. Based on its inducible expression profile under salt stress, *Os05g0349800* is proposed as the most promising candidate gene underlying *qSTS5*.

## 5. Conclusions

This study utilizes a population separated by DY80 and R974. Through preliminary mapping and validation analysis, a major QTL *qSTS5* controlling salt tolerance during the seedling stage was identified. By analyzing RNA-seq, sequence alignment, and encoding products, an annotated gene *Os05g0349800* was selected as the most likely candidate gene. This study provides a theoretical reference for elucidating the mechanism of strong salt tolerance in Dongxiang wild rice. In addition, the identification of *qSTS5* offers an excellent gene resource for improving salt tolerance at the seedling stage.

## Figures and Tables

**Table 1:** Descriptive statistics of salt tolerance at germination and seedling stage of BIL and F_2:3_ population.

Population	Traits ^a^	Minimum	Maximum	Mean	SD	Skewness	Kurtosis	Mean ± SD	
								Female	Male
BILs	STG	0.0	98.3	58.6	24.1	−0.3	−1.0	36.7 ± 4.7	99.2 ± 0.5
	STS	0.0	82.5	33.1	18.2	0.4	−0.3	15.3 ± 4.1	90.1 ± 2.2
F_2:3_	STS	0.0	87.5	25.39	19.05	−0.1	0.8	18.3 ± 3.1	85.9 ± 2.2

^a^ STG, salt tolerance at germination stage; STS, salt tolerance at seedling stage.

**Table 2:** Primary mapping for STG and STS using the BIL population.

Traits ^a^	QTL	Chr	Interval	Physical Position	*LOD*	*A* ^b^	*R*^2^ (%) ^c^
STG	*qSTG2*	2	CHR2-15.8–RM71	5,507,312–8,761,504	2.9	9.7	7.5
	*qSTG5*	5	CHR5-0.7–5-1458	710,658–1,457,663	3.4	8.7	6.3
	*qSTG9*	9	CHR9-17.1–9-6118	4,354,691–6,118,061	4.0	−9.4	8.6
	*qSTG10*	10	CHR10-70.8–CHR10-82.6	14,723,136–17,474,848	2.7	11.5	5.1
STS	*qSTS2*	2	CHR2-101.9–RM240	28,417,539–31,448,128	2.7	6.8	3.9
	*qSTS5*	5	CHR5-50.7–CHR5-86.0	17,327,039–19,258,988	8.0	9.8	14.8
	*qSTS6*	6	RM204–CHR6-14.7	3,168,425–5,300,255	2.8	11.3	7.2

^a^ STG, salt tolerance at germination stage; STS, salt tolerance at seedling stage. ^b^ *A*, additive effect of replacing a DY80 allele with a R974 allele. ^c^ *R*^2^, Proportion of phenotypic variance explained by the QTL effect.

**Table 3:** Genetic effect validation of *qSTS5* using the F_2:3_ population.

QTL	Interval	Physical Position	*LOD*	*A* ^a^	*D* ^b^	*R*^2^ (%) ^c^
*qSTS5*	CHR5-50.7–CHR5-86.0	17,327,039–19,258,988	10.4	12.0	2.5	18.5
*qSTS12*	CHR12-43.1–CHR12-84.4	8,025,081–18,310,512	3.1	7.0	−1.8	5.2

^a^ *A*, additive effect of replacing a DY80 allele with a R974 allele. ^b^ *D*, dominance effect. ^c^ *R*^2^, proportion of phenotypic variance explained by the QTL effect.

**Table 4:** Sequence comparison of the eight DEGs in the coding region.

Locus Name	Polymorphism Using R974 as Reference ^a^							
*Os05g0331900*	NP	no difference						
	AP							
*Os05g0341450*	NP	no difference						
	AP							
*Os05g0349800*	NP	G 140 T	G 141-					
	AP	G 47 V	-					
*Os05g0361700*	NP	A 50 G						
	AP	H 17 R						
*Os05g0373900*	NP	A 234 G	T 252 C	A 276 G	T 378 C	C 459 T	A 465 G	G 468 C
	AP	A 72 A	Y 84 Y	L 92 L	C 126 C	N 153 N	T 155 T	L 156 L
	NP	T 471 C	T 474 C	T 480 G	C 579 G	T 621 A	T 624 A	C 666 T
	AP	Y 157 Y	G 158 G	L 160 L	R 193 R	L 207 L	A 208 A	I 222 I
	NP	T 669 C	T 681 C	C 687 A	A 804 C	A 819 C	C 1008 A	A 1026 G
	AP	A 223 A	L 227 L	G 229 G	A 268 A	A 273 A	K 336 N	T 342 T
	NP	G 1105 A	C 1110 T	G 1138 A	T 1176 C	G 1242 A	G 1258 A	T 1275 C
	AP	I 369 V	I 370 I	T 380 A	F 392 F	L 414 L	I 420 V	D 425 D
*Os05g0381400*	NP	no difference						
	AP							
*Os05g0390300*	NP	T 67 C	T 228 C	C 234 G	G 271 A	C 398 A	A 442 G	
	AP	L 23 L	G 76 G	A 78 A	G 91 S	A 133 E	S 148 G	
*Os05g0399400*	NP	T 53 C	T 165 C	T 189 C	A 207 T	T 210 C	C 216 T	T 243 G
	AP	V 18 A	G 55 G	D 63 D	G 69 G	G 70 G	G 72 G	G 81 G
	NP	C 354 A	A 360 C	A 396 C	G 547 C	G 639 A		
	AP	F 118 L	T 120 T	G 132 G	A 183 P	G 213 G		

^a^ NP, nucleotide polymorphism; AP, amino acid polymorphism.

## Data Availability

The original contributions presented in the study are included in the article/Supplementary Materials, and further inquiries can be directed to the corresponding authors.

## Supplementary Materials

The following supporting information can be downloaded at https://www.mdpi.com/article/10.3390/biology15090702/s1: Figure S1. Distribution of the seven DY80 chromosomal fragments on the DW14 genome. The two red markers added to the secondary mapping population were used to narrow down the *qSTS5* region; Table S1. Primer sequence for qRT-PCR; Table S2. Primer sequence for sequence analysis; Table S3. Primer sequence for genetic map construction; Table S4. Summary of RNA sequencing and alignment data for each sample; Table S5. The FPKM value of deferentially expressed genes in *qSTS5* region.
