# Supplementary material for: Identification and Validation of qSTS5, a QTL Associated with Salt Tolerance at Seedling Stage in Dongxiang Wild Rice
Source: Biology (Basel). 2026 Apr 29;15(9):702. doi: 10.3390/biology15090702 (PMC13162581; doi:10.3390/biology15090702)
Supplement: Supplementary file 1 [file biology-15-00702-s001.zip › biology-4253451-supplementary.pdf]

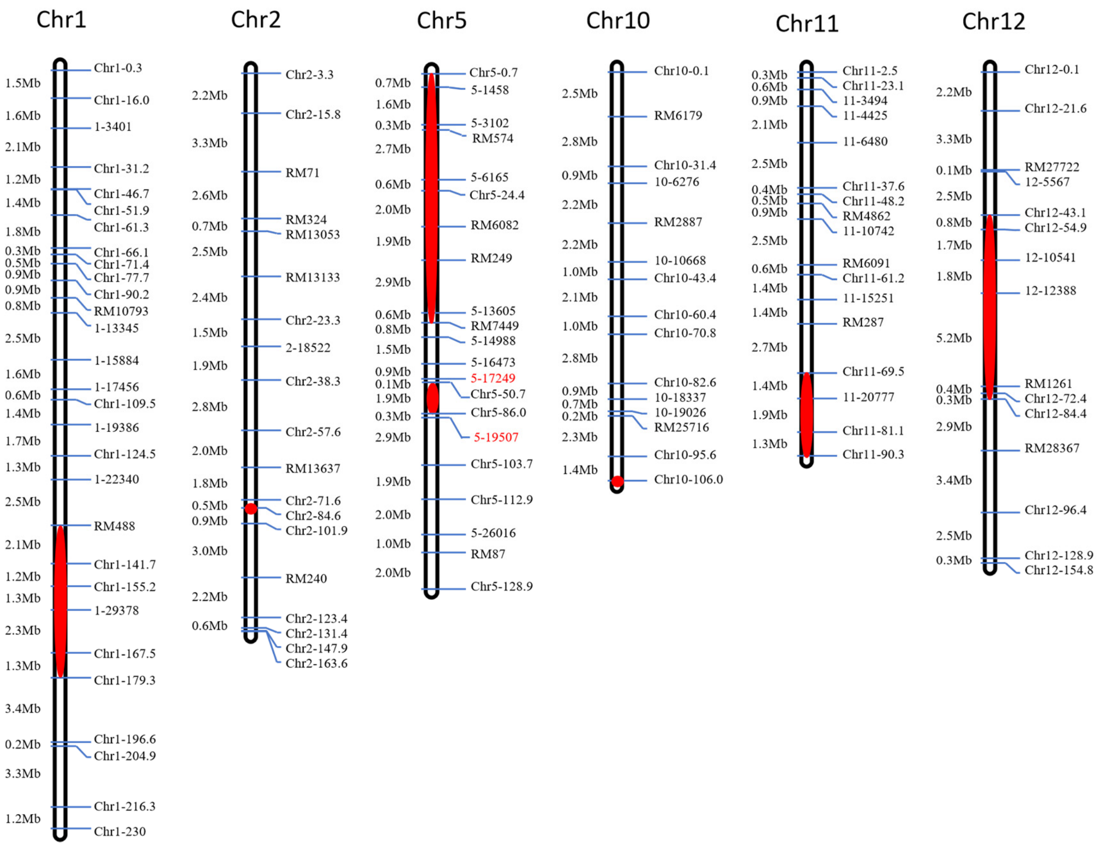

Figure S1. Distribution of the seven DY80 chromosomal fragments on the DW14 genome. The two red markers added to the secondary mapping population were used to narrow down the *qSTS5* region.

Table S1. Primer sequence for qRT-PCR

Primer name	Primer sequence (5' - 3')
Os01g0348900-F	CCCAAGAAGCTGTTAGGCGT
Os01g0348900-R	CTGTAGAGGTGCCTTCGCC
Os01g0501800-F	TTGAGCGTCACCAAGAGCAA
Os01g0501800-R	TGACATCCTTGGGCACCTTG
Os01g0600900-F	TCGTCGACCCGCTCTACC
Os01g0600900-R	CGGCCGTTCTTGATCTCCTT
Os01g0639900-F	ACATGGTCCCAGCTTACTGC
Os01g0639900-R	GAGGAGGGCCTTGATTCCAC
Os02g0161900-F	GCCAAGATCCAGGACAAGGA
Os02g0161900-R	CATACCACCACGGAGACGAA
Os03g0592500-F	CAGAGCATCCTCGCCATCTG
Os03g0592500-R	CCTGGGTACACCTTGTCGAG
Os06g0101600-F	GCGAGACCATCACGTTCAAG
Os06g0101600-R	TTGAGGTACTCCTCCTGCGAT
Os08g0200300-F	GTGGGAAGAAGATCAAGACCGA
Os08g0200300-R	GGTACACACCCTTCCCCGT
Os11g0707000-F	CTTCTACATCGCCCCTGCTTT
Os11g0707000-R	AGATACCCAGGATGAGTGGGA
OsActin-F	CTTCATAGGAATGGAAGCTGCGGGTA
OsActin-R	CGACCACCTTGATCTTCATGCTGCTA

Table S2. Primer sequence for sequence analysis

**Primer name**	**Primer sequence (5' - 3')**
Os05g0331900-F1	TGGTACTCTGGCAGCCACAA
Os05g0331900-R1	GCTCATTGTTGCATACGATCGG
Os05g0331900-F2	CGATCTGAAGGCCCGTAGTA
Os05g0331900-R2	AGTGGGTTTCTCCTTATGGG
Os05g0341450-F1	CCCTCACTCTAGGGATTGCTTTG
Os05g0341450-R1	GCTCGGGTTTCATCGAATCC
Os05g0341450-F2	GTTGGCCAAGCAAACCACTT
Os05g0341450-R2	GTGATCTTTCGGAGTTGCGG
Os05g0349800-F	CTATAAGAAGGACAGCGCGG
Os05g0349800-R	CCTTCTCCTGTGTTTTGCGC
Os05g0361700-F	CTGTGCCAATGTACTACTCCTACC
Os05g0361700-R	TCCATCGGATGGGACCTAACGC
Os05g0373900-F1	TCTTGGACGACGTGATCTCC
Os05g0373900-R1	TCGGGAGACCTGATCACGAG
Os05g0373900-F2	GATCAAGGCACTTGATGCTG
Os05g0373900-R2	TTTCAGTGGTCGAGTGCAAC
Os05g0381400-F	GTCGCGCCTCTCCGGAAATCA
Os05g0381400-R	GTACTCACATGCTGCAGTCATACGG
Os05g0390300-F	CCTCCTTGTGTGGCCCTATA
Os05g0390300-R	TGAAGCAAGTTGACGCAGCA
Os05g0399400-F	AGCCGTCTATAAAAGGCAGG
Os05g0399400-R	GACACACCATACTTCGACTCG

Table S3. Primer sequence for genetic map construction.

Primer name	Chr	Physical distance	Distance（bp）	Forward primer	Reverse primer
chr1-0.3	1	256,208		ATTGACAATTGTTGA AGCGAGAAAAGGG	CCTCCAGGGTCTTCG ACCGCCAGT
chr1-16.0	1	1,765,245	1,509,037	AGGGAGTTGGCTGCG ATGCTTCTT	TTGGAGAGGTTGAGG TGGGTGAGCGA
1-3401	1	3,400,615	1,635,370	CAATGTAGACGAATC AGCGATGCA	AATGTAGACGAATCA GCGATGCC
chr1-31.2	1	5,498,833	2,098,218	GCCTAAGACCCAGTG ACTT	GGCATTTCACAGCTA GACTTTCC
chr1-46.7	1	6,677,126	1,178,293	CCTGAAAGCCACATC ACAAAGT	GTACTTTCCTTAGCCC GGGG
chr1-51.9	1	6,693,228	16,102	CATGACCCGGGAGAT GAACC	CCGCTTGCTCCATACC TGAT
chr1-61.3	1	8,075,234	1,382,006	CAATTTCCATAGGCT GCATG	GCTTGGGTTAGCGAC GAC
chr1-66.1	1	9,866,595	1,791,361	CGGTTAATGTCATCTG ATTGG	TTCGAGATCCAAGAC TGACC
chr1-71.4	1	10,215,898	349,303	TCTTTGTGCACATCTA C	GAATGTTCACTTCAA TTGCCAGTT
chr1-77.7	1	10,714,089	498,191	CGAGCCATCACAACC CTAA	ACTTCCTCGATGCTG ATAGATCC
chr1-90.2	1	11,622,738	908,649	GCAAATACTGACCAC ATTA	AGCTCGTGAGAGAAG AAAAAAGG
RM10793	1	12,553,830	931,092	GACTTGCCAACTCCTT CAATTCG	TCGTCGAGTAGCTTC CCTCTCTACC
1-13345	1	13,344,678	790,848	AAATTCGAATTAGAT CAACAAGGACTACT	CGAATTAGATCAACA AGGACTACC
1-15884	1	15,883,941	2,539,263	AGTCCTTCTGCTTTGT GTCACTC	GAGTCCTTCTGCTTTG TGTCACTT
1-17456	1	17,456,083	1,572,142	GCACGTCCCTCATTCT CTCAG	ATATGCACGTCCCTC ATTCTCTCAA
chr1-109.5	1	18,029,855	573,772	CAGCGCGGAGAGAG GATA	AGCTCTCGATCCCTA ACAC
1-19386	1	19,385,818	1,355,963	TGCTACTTGAGTTGCA TCTTCAAACT	GCTACTTGAGTTGCAT CTTCAAACA
chr1-124.5	1	21,057,423	1,671,605	AGTCCACCCAGTCCG TCAATGG	GAACACTCCTCCTCT GTTCTCACTCG
1-22340	1	22,339,942	1,282,519	CCCGGTGTGATCTGC GGC	CCCCGGTGTGATCTG CGGT
RM488	1	24,804,715	2,464,773	CAGCTAGGGTTTTGA GGCTG	TAGCAACAACCAGCG TATGC

Primer name	chr	Physical distance	Distance（bp）	Forward primer	Reverse primer
chr1-141.7	1	26,912,440	2,107,725	CGACAGACCAAGAATGGATA	ACCGGTGCAATGTTTAGTT
chr1-155.2	1	28,108,330	1,195,890	AGAGTATATGACATGGAGTAAA	CATTTTCACTATAGTCTGGGCTC
1-29378	1	29,378,022	1,269,692	CGTTTGACCCTATCCTACGCC	CCGTTTGACCCTATCCTACGCA
chr1-167.5	1	31,709,475	2,331,453	TCTTCTGCCGCTTAACTATACAA	GTAGAGCAGTGATGAAAACCT
chr1-179.3	1	33,054,572	1,345,097	CACCCATTTGTCTCTCATTATG	CCACTTTCAGCTACTACCAG
chr1-196.6	1	36,498,439	3,443,867	TTCTGATCTCACCCGGATTC	AGATGGAGATGGCGATGAAG
chr1-204.9	1	36,735,178	236,739	AGTCAGCTCACTGTGCAGTG	GAGGTACTTCCTCCGTTTCAC
chr1-216.3	1	40,016,503	3,281,325	ATCCCAGAAGCAAACTCGAA	GGGGTTCGTGAGTTTGTCAG
chr1-230	1	41,198,359	1,181,856	TAGACAAAGCAACGGGTTCC	CGGAAGCAGGAGAATCGTAG
chr2-3.3	2	3,280,974		GATGCATCACTTGGCTAGGC	TGCTGCATCCCATGGTAGTA
chr2-15.8	2	5,507,312	2,226,338	GAATGTCAAGGTGAAAGGAGGCA	AGACAGGGTTCAGGTTGGCTTTG
RM71	2	8,761,504	3,254,192	CTAGAGGCGAAAACGAGATG	GGGTGGGCGAGGTAATAATG
RM324	2	11,388,913	2,627,409	CTGATTCCACACACTTGTGC	GATTCCACGTCAGGATCTTC
RM13053	2	12,090,334	701,421	TTTCTGGCGACGTGATTTGTCG	CAATTCGGAAGAGCAAACATGACC
RM13133	2	14,609,111	2,518,777	GAGGATTTCCGTGGGAATTATGC	AGTCAACAGCGAGATCAGTTAAGAGG
chr2-23.3	2	17,003,027	2,393,916	GAGCAAATGGGTTGTTTGAGAGG	GGCTCTCTTACTTTCTCCTTCATGG
2-18522	2	18,521,952	1,518,925	GGTGTGTGCTTAAGGTGGCTAT	GGTGTGTGCTTAAGGTGGCTAC
chr2-38.3	2	20,404,556	1,882,604	CCTCACGATTTTCCTCCAAC	ACGGTGGGATTAGACTGTGC
chr2-57.6	2	23,232,361	2,827,805	GCAAGAGGAAGCAAGTCCAC	CTGATTCGACGGGACACAC
RM13637	2	25,274,640	2,042,279	CTCAACTTGCACCACCAAACG	GCCACTGTTTCCATCTCCTAGC
chr2-71.6	2	27,072,716	1,798,076	CGCCTTTCCACATTCCTGGCTCTT	AGCGTTCGTCGTGTCCCATCTCG

Primer name	chr	Physical distance	Distance（bp）	Forward primer	Reverse primer
chr2-84.6	2	27,538,266	465,550	GTTTCAGACTTTCAGCCGTTCGC	TAGGCACCTGCTTCATCAATACA
chr2-101.9	2	28,417,539	879,273	AAAGTGGGTTTCTTGATTTGGTTAG	AAGACCAGTATGCCACAAGAAAG
RM240	2	31,448,128	3,030,589	CCTTAATGGGTAGTGTGCAC	TGTAACCATTCCTTCCATCC
chr2-123.4	2	33,682,808	2,234,680	TTGTGGTGGCCGTACTGATA	CCTAGCTGCTCCTGCACTCT
chr2-131.4	2	34,287,673	604,865	GCAAAACCTCCCAGCGAAAA	GGGTCAAGAAAGCTGTGGGA
chr2-147.9	2	34,444,317	156,644	TTTGACCTCAACTCCCAAGC	GCTCCCTCACTCACAACTCC
chr2-163.6	2	34,466,028	21,711	AACTGGCTGTACTGTTGTCATCT	AGCCCATAGTAAGTACATCACAGTTTT
chr3-1.9	3	1,909,161		TGTCCCCTCTAAAACCCTCC	GACATTCTCGCTTGCTCCTC
chr3-5.3	3	4,095,733	2,186,572	GCAGAAACTTTTGGTTACATAAA	GCATAGAAGGTAGACAGTCAGAA
chr3-8.5	3	5,906,887	1,811,154	TAGAGTCGTTCGTTTATTTGTGC	GGGTGAGATGTTTTGCTTTGTTT
chr3-13.7	3	6,804,948	898,061	TCTTGCTCTTTCCCCGACCCGTG	CAAGGAGGGCGTCACGAAGAT
chr3-18.7	3	8,198,400	1,393,452	TGACAAGCAGATTGGACTGG	CTCACACGCCTCAGGTGTAA
chr3-30.0	3	8,439,737	241,337	GGTCAAGTCTAGTTAGCAAGAGC	CCTTGATTATTGAACGTCCTCTT
chr3-43.3	3	9,832,662	1,392,925	AATGATCACACACTCTGCTCTCAGC	GCACGATCTTCCTCTTGATGTCG
RM14909	3	12,688,495	2,855,833	CACCGCCATGAACTCCTACTCC	ACGCCGAAGTCGAACTGGTAGG
chr3-48.8	3	14,622,466	1,933,971	AAAGTATTGTATTACCATTCCTG	CTGCTAAAATCTTCATA
3-16339	3	16,339,348	1,716,882	GCCCTGCATATGTATCGCTAGCT	GCCCTGCATATGTATCGCTAGCA
RM15236	3	17,713,684	1,374,336	CACTCCCTCCTCTCTCCTCTCC	GTTGGTTGGTCGGTTGCTTACC
RM15290	3	19,242,432	1,528,748	TACTCTCTGCTTCCAAGTTGTTGC	GGGTTGGTATTAATGGGAGTGG
3-20411	3	20,410,629	1,168,197	ACAAAAGAATGGACTAGTTTGGCTGTA	CAAAAGAATGGACTAGTTTGGCTGTG
3-21107	3	21,107,337	696,708	TCTCTTACATCTTGCCAGACTCGT	CTCTTACATCTTGCCAGACTCGC

Primer name	chr	Physical distance	Distance（bp）	Forward primer	Reverse primer
chr3-58.7	3	22,168,490	1,061,153	GCAGCACCAGGGTTC TCACC	CTTCCATTCCTTCCAC CTCTTCC
chr3-68.6	3	23,886,673	1,718,183	TTGGACAGCCTGGAC AGTCATAC	GCAATCCTAAAACCT CCAAATAA
chr3-88.2	3	25,055,391	1,168,718	TCTTGCTCTTCTTCAC TGGACTG	TTCAGAGGGTACACT GTGGATGG
chr3-106.7	3	26,705,468	1,650,077	CCTACTACTACTTTCT TTGG	ACAGTACCTTCTTTGC ATCTCCC
RM15717	3	27,677,118	971,650	GCGTTTGTAGGATGT CATGTGG	CAGGTCGGTTTCCTTG TAGTTCG
3-29311	3	29,311,404	1,634,286	CATCAGCAGATGCAG CACAGG	CATCAGCAGATGCAG CACAGC
chr3-111.7	3	30,677,202	1,365,798	TTCACACGCATCGAG TAAGC	AAATCGTCGAACACC TCTCG
chr3-121.4	3	33,003,423	2,326,221	GAAGCAGAGGTAATG CCCTAAAA	CATCAGGGAAACAG AACAGACCA
chr3-130.6	3	33,431,609	428,186	CTGACTCACAAGACA ACAGGG	CATAAACCGCTCGGC ATTG
chr4-0.2	4	197,708		CTAAGCAAGGATTTT AACAACTT	TAGCACGAAACAAG GGGAGGATA
chr4-17.4	4	689,354	491,646	GTACACACCCACATC GAGAAG	GCTCTATGCGAGTAT CCATGG
4-2485	4	2,484,571	1,795,217	GGACAAGGCAATGC AGGTCACA	GACAAGGCAATGCA GGTCACG
RM16408	4	3,826,798	1,342,227	CGTCGAATCGATAGA GCGACTTAGG	CTTGCATGGTCAGAC AACATCTCC
RM261	4	6,558,964	2,732,166	CTACTTCTCCCCTTGT GTCG	TGTACCATCGCCAAA TCTCC
RM16563	4	9,555,191	2,996,227	CACCATCTTCATCTCC ACCTTCC	TCCTCCTAATCCACCC ACAGAGC
4-11880	4	11,879,947	2,324,756	TAGTGGTTCGGTTGA CTGTAAGAATT	AGTGGTTCGGTTGAC TGTAAGAATC
chr4-32.4	4	13,163,462	1,283,515	TGGAACAGATAGGGT GTAAGGG	CCGTTCACAACACTA TACAAGC
chr4-42.8	4	16,600,984	3,437,522	CGAGGTTTGAACAGA CATATTAGA	TAGTGCTACCTCCAA ACAAGGGT
4-17907	4	17,906,798	1,305,814	CAAGCTGTTGTAAAA GGTACACCG	CCAAGCTGTTGTAAA AGGTACACCA
RM1359	4	19,686,510	1,779,712	CGACTTGCCAAAGGT CAACG	GATTCTACGGGCCAC AAGTCC
RM3463	4	20,128,692	442,182	TGCCTCTCTACCGCA ACACAGC	CCCTTCCCTTACCTCT CCATTCC

Primer name	chr	Physical distance	Distance（bp）	Forward primer	Reverse primer
4-20911	4	20,910,701	782,009	CCGCGTCACCGTGTT CCAT	CCGCGTCACCGTGTT CCAC
chr4-58.0	4	23,218,253	2,307,552	ACTGATGCCGCCAAG TGTAGGG	CCCGAATTGCTTTAA GCTTCTGTACC
chr4-64.2	4	24,574,499	1,356,246	GCAGCATGTTTATTG ATTCTTTGAG	ACTTCCGACTTAGTTC GTTGGTG
RM470	4	27,898,526	3,324,027	CCCTCCCGTAGACCTT GTACCC	CCACAGCTAACCAAT CCTTCTCC
chr4-74.8	4	29,076,296	1,177,770	GCAATGTCAGTTCCT GATTTGT	AAAGGACGAGCACA ACATACCC
chr4-87.3	4	29,962,862	886,566	GCTCCTATTACAACCT ACTCCTG	TAGATGTATGGGTCC GCTTTAGT
4-31477	4	31,476,593	1,513,731	GGTTCTCAACAGCAC GGTAG	CTGGTTCTCAACAGC ACGGTAC
chr4-103.0	4	33,116,030	1,639,437	AGCCAAGGTAGATAG GGTTC	GGCAGTCTCGTTGGA GTC
chr4-110.1	4	33,848,468	732,438	GCAGCAATACACGAA AAATCACCGA	CTCTTCCATTAGCCTC CTTTT
chr4-117.9	4	34,932,202	1,083,734	GGTGCTAGCCTGGGG TTTAT	CACTTCACACGCCCA AACTG
chr5-0.7	5	710,658		TGGTTATTTGTTATTTT AGTTGGGTG	TAGACTAGAGTTGGA GACG
5-1458	5	1,457,663	747,005	ACTAATAACTCAGGC TAAGCTATTGAT	ACTAATAACTCAGGC TAAGCTATTGAC
5-3102	5	3,101,601	1,643,938	GTGAACATCCCGCTG CAACTG	GTGAACATCCCGCTG CAACTC
RM574	5	3,419,526	317,925	AAACTAGCCACGGTT TGGTAGGG	AGGGTGGCAGGGATG TAATTTCC
5-6165	5	6,165,472	2,745,946	GGTGCACTGCGAGGG CATC	CAGGTGCACTGCGAG GGCATT
chr5-24.4	5	6,775,162	609,690	CAGGTTGCGAAGAGT GTTGAAGA	GTCAGTGGGTCGTTTT TTGCTAT
RM6082	5	8,799,566	2,024,404	GCGGGTGCTTATGTC ACCTACACG	AATGAGGGTGGGAGC GATGTCC
RM249	5	10,656,583	1,857,017	CAACTCCACTCCAGA CTCAACTCC	GGTATGATGCCATGA AGGTCAGC
5-13605	5	13,605,325	2,948,742	TAGAAATTATGTTTA ATGAAATGATTTGAT AT	AGAAATTATGTTTAA TGAAATGATTTGATA C
RM7449	5	14,164,624	559,299	GCAGTGACCGGAGCT TACATAGC	GCTCGATCATATGGC TGCAAGG

Primer name	chr	Physical distance	Distance（bp）	Forward primer	Reverse primer
5-14988	5	14,987,870	823,246	TTATCTCTACCAAGGTTTACGATTT	CTTTATCTCTACCAAGGTTTACGATTC
5-16473	5	16,473,206	1,485,336	GACAAGGTGGACGATGATCGACT	ACAAGGTGGACGATGATCGACG
chr5-50.7	5	17,327,039	853,833	TGCAAACCCACTCCAACACT	CGAGTGTGCATGCAATATGGC
chr5-86.0	5	19,258,988	1,931,949	TCTTGCCCGTCACTGCAGATATCC	GCAGCCCTAATGCTACAATTCTTC
chr5-103.7	5	22,125,940	2,866,952	TACTGCTATCGCTAAACATGAGA	CTCCAGCACTGAACATATCGTGAAGCA
chr5-112.9	5	24,049,502	1,923,562	AATCCTGCCGTCCATCTTCA	ACAGAGGGAGAATTATGTTTGAT
5-26016	5	26,015,789	1,966,287	GGATACGGTTTAGAATTGAGATCCTC	GGATACGGTTTAGAATTGAGATCCTT
RM87	5	27,020,964	1,005,175	CCTCTCCGATACACCGTATG	GCGAAGGTACGAAAGGAAAG
chr5-128.9	5	29,060,729	2,039,765	TCAGTAGCATAAAGGCACAGGAT	GCAACATAGGAAACTATACGGTCAT
chr6-0.2	6	227,513		GCAGGTAGTAGGTTGGTAGGTGG	TACAGTCCCACCCTAATGAATAC
RM204	6	3,168,425	2,940,912	GTGACTGACTTGGTCATAGGG	GCTAGCCATGCTCTCGTACC
chr6-14.7	6	5,300,255	2,131,830	CCAGGCGCTCATCTACAAGT	ATGTACGTACGGTGATGGGG
chr6-17.2	6	7,213,877	1,913,622	ATTCGGTGTATTGTTGCTCG	ATGGAAGTCAATCCTCCCTACAC
6-9007	6	9,007,314	1,793,437	CATTCAGCTCTGCGGTCGATG	CATTCAGCTCTGCGGTCGATC
RM3330	6	10,907,997	1,900,683	ATTATTCCCCTCTTCCGCTC	AAGAAACCCTCGGATTCCTG
6-12353	6	12,353,277	1,445,280	AAAATATGCAGAAAAGCGAAGTAAACACAA	ATATGCAGAAAAGCGAAGTAAACACAG
chr6-26.9	6	13,979,642	1,626,365	TGAATGCAGGACTAGATGAA	CCACCCTCAATTATATTCCA
RM20059	6	16,066,065	2,086,423	CTGGTAAAGGACCTGTGGTCATGG	TCCGATAATCAAATCGGGATGG
chr6-34.7	6	17,953,681	1,887,616	ACCCTGGAACTAATAGCATA	ATGTTCATCATTGCGTTTTGTT
RM7193	6	19,910,922	1,957,241	ATGTGGGAATTTCTAGCCCC	CCCTAGTTTTCCAAATGGCC

Primer name	chr	Physical distance	Distance（bp）	Forward primer	Reverse primer
RM1340	6	22,960,633	3,049,711	ATCGATCTCCACCAC TTCCTTCC	CCCTACTCCCAGTAA CCCAAATAGG
chr6-50.0	6	24,234,137	1,273,504	CAAGAATCAAGAGCC GAGAGTCC	TCTTCTGTACGGTTTC TTGGTTGC
chr6-71.9	6	25,203,289	969,152	CCTGGTAAAGAAGAG CCAATGAT	CTTGACTATCTTGGGA CTGTGAG
chr6-95.3	6	27,094,497	1,891,208	CCGCCTTTCCCTATCT CTCT	TCCTACCACCTCCAA TGTCC
chr6-103.8	6	28,145,544	1,051,047	GAGGACTTGGCGTCT TAGAT	CCAACTGTGGAAACC ACTAA
chr6-137.4	6	30,043,832	1,898,288	ATTCTTCCAAAATAG ACCGTAGG	TCCGAGATCAAAATC CAATG
chr7-0.9	7	903,040		GTAGCTCCATGCCAG TTTGTGG	AACCTTCTTGATTGGC CATCTCC
chr7-21.1	7	3,146,214	2,243,174	TCTCACAATCGTCGTC CGCATCCTC	CGTGCTTGGCGATGC TGCTAATGC
chr7-25.2	7	5,200,751	2,054,537	CAAGAGGAGCGGCTG TCTGTGG	ATGCTTGTAGGCTTTG GAGATGG
RM5672	7	6,413,197	1,212,446	ATATAGAGGCAACCA CTTAGCC	TACACCCTACAAGGA AACAAGC
chr7-30.0	7	9,002,950	2,589,753	CTCTGGATTTGGAGG AATTA	AATGGAACAAACGCT CCTA
7-11874	7	11,873,548	2,870,598	CCACCCAATGAAAGT AAAGCATTTTG	AATCCACCCAATGAA AGTAAAGCATTTTT
chr7-39.8	7	12,450,421	576,873	AGACGTCAAGCAGAT GGTA	GATGATGGCTGTCAT TCTAA
7-13281	7	13,280,855	830,434	GTCCACCAGCTCGGT GCCTT	CCACCAGCTCGGTGC CTG
7-17500	7	17,500,485	4,219,630	GGAGCAGGAGATTCC ACAGAG	CGGAGCAGGAGATTC CACAGAA
7-18257	7	18,256,827	756,342	CCTGCATTGTTACCTG ATCAAGTC	CCCTGCATTGTTACCT GATCAAGTT
RM11	7	19,204,350	947,523	ATCGGTGCTTGGCTG GATAGC	CCACCTTCTTCTCCTC CTCTTCC
chr7-46.4	7	20,763,512	1,559,162	ATTATCGGTGGTTAG ATGGAA	CCACGATGCGTCGAA CCAGTG
7-22593	7	22,593,213	1,829,701	ACATACAGAACTTAT AGGGATTAACAAGT	CATACAGAACTTATA GGGATTAACAAGC
chr7-57.3	7	23,985,483	1,392,270	CTCCTCCCACGTCAA GGTC	ATCACCTGAGAAACA TCCGG
RM22065	7	27,080,800	3,095,317	GAAGTTAATTGAGCT TGGCGATGG	AGCACGTGGAACAG AAAGAAAGG

Primer name	chr	Physical distance	Distance（bp）	Forward primer	Reverse primer
RM22174	7	29,477,616	2,396,816	CTGAGGTTTGCCGAC GAGTTCC	GGGCCATTCTTTCCTT GGTTGG
chr8-0.7	8	683,990		AAGGAGAAGTTCTTC GCCCAGTGC	GCCCATTAGTGACTG CTCCTAGTCG
chr8-14.4	8	1,707,188	1,023,198	AGCTTGTATGTGTGC ACGGA	TCCATGGATTGTTGAC GAGGA
chr8-43.8	8	5,240,731	3,533,543	AAGGGTATGATACAG AATAAGTGATGG	CAAGGTGGAAGGGC ATTGTGGAT
chr8-57.1	8	7,762,076	2,521,345	GAAGGAAGGGCGGG TTAGTACGG	CACCGCGGATATTAC CACCTTCC
8-9071	8	9,070,836	1,308,760	AGGCTCAAGAGAAG CTTCTTTCTG	AGGCTCAAGAGAAG CTTCTTTCTC
chr8-67.2	8	10,798,107	1,727,271	TGGGTCCCTATGCAT GTGAG	GGTTGATGAGTCTTG ATGTGCA
RM22834	8	12,284,753	1,486,646	GACATTGCCCTCATG GAGATGG	ACTCCTCGTGGACAT CATCAACC
8-13817	8	13,816,706	1,531,953	CAATGCCTCAAAGGC AGCACG	CCAATGCCTCAAAGG CAGCACA
RM22899	8	14,762,955	946,249	TTGCTGTAATGCTGTT CCATCC	CGAAGGCGACCTTTC TAGTCG
chr8-72.0	8	17,945,060	3,182,105	GTAATCGATGCTGTG GGAAG	GAGTCATGTGATAGC CGATATG
chr8-80.0	8	18,987,440	1,042,380	CCCTTGGATCATATCC TTTTCTT	AAGAAGAAAGTTGTC GGGCAT
chr8-90.8	8	20,644,265	1,656,825	TATACAATGGGTACA CGGCTTCA	GTGCCACTAACCTCTT ACACCTCAATA
chr8-109.4	8	23,117,196	2,472,931	CAACCAGGAACAGTA ATGGAAAG	TTAGCGGCGGATTTG TCGTC
chr8-120.2	8	24,273,349	1,156,153	GACAGGGAGTGATTG AAGGC	GTTGATTTCGCCAAG GGC
chr9-3.8	9	3,803,711		TTGCACAAGAGGCAA CACTC	ATCGAACCATCCAGG ATGAC
chr9-17.1	9	4,354,691	550,980	GCATCTCAGCAAACA AGAACAACC	GAGGCCATCAAGCAG TTCTCC
9-6118	9	6,118,061	1,763,370	GGGGTTTGGAAGAGC TAGAGTC	GGGGTTTGGAAGAGC TAGAGTA
chr9-29.9	9	7,888,511	1,770,450	CAATGCGAATCAAGG TTCAGAGC	GTGAGGTGGTGGGTG GTTATGG
RM1328	9	9152293	1,263,782	GAATGGGATTAGACG ATTTG	CCATGAGTGACATCA AAAGG
9-9497	9	9,497,051	344,758	CGACGTCGTGGTCTT AAATTTTGTC	ACGACGTCGTGGTCT TAAATTTTGTA

Primer name	chr	Physical distance	Distance（bp）	Forward primer	Reverse primer
9-10857	9	10,856,832	1,359,781	TCAGCAACTTGGCCCTTCTATCAT	CAGCAACTTGGCCCTTCTATCAC
chr9-50.0	9	11,928,947	1,072,115	CAACCACATGGAAATTGTGC	GGAGGGGGTTGTGATTTACC
chr9-61.6	9	12,212,506	283,559	TCGTAACCAATCAACCAGGG	TGCTGGTAGGGTTCATAGTG
chr9-69.2	9	13,164,222	951,716	GTCTTTCTTCCTGAACAATG	ACACCATTAGACAGTTGAGG
chr9-78.9	9	14,567,027	1,402,805	GAACAGAGGAGGAGATCGAGAGG	CTTCTTGGGAGATGCAGAAATGG
chr9-82.3	9	15,065,031	498,004	GACGAGACCTCCCCAGATCT	CATCCATCCACGACACACCA
chr9-98.5	9	16,752,256	1,687,225	GTCCATGCCTGTGGGAACACG	CCGACGGCTATGTCATCAACTGC
chr9-115.0	9	18,864,743	2,112,487	CTGCTCCAATCAATTAAACTTAT	GTTATGATTAGGTGCTGATTGAC
chr9-119.1	9	19,788,697	923,954	TGCCAGCCCAAATCAGGAAA	TGAGCAGAGACACAGGCAAG
chr10-0.1	10	54,866		GTCACGTGGCGATTTAGGAG	AGATGGTTGCCAAGAGCATG
RM6179	10	2,566,362	2,511,496	ATCTCGTCCATCTCCGGC	TCCAACGGTCAAGATTAGCC
chr10-31.4	10	5,347,005	2,780,643	TGCTGTACTCCCCAACTAAAT	TTGGCAAGTATTGGTAGCAGA
10-6276	10	6,276,084	929,079	GCTTGTACCTGCAGCTTGAG	CCTGCTTGTACCTGCAGCTTGAA
RM2887	10	8,514,056	2,237,972	GATCAATATGATTTTTTTCA	TAGTCGATTACTATTGGGTA
10-10668	10	10,667,565	2,153,509	CACAAGAAAATTTGTAGTTCATCAACAAAC	CCACAAGAAAATTTGTAGTTCATCAACAAAT
chr10-43.4	10	11,644,578	977,013	TGGTTTAAGCACCGGGTTTTACG	AGTGGATTGATGATAACTTGTTGCTTT
chr10-60.4	10	13,705,257	2,060,679	TCTGAAATACCTGAATCTGAATG	TGAATTACGCAAACAGAGGA
chr10-70.8	10	14,723,136	1,017,879	TGGCAGCTTCATCCACTTGT	AGCAGTACACAAACAGGCGT
chr10-82.6	10	17,474,848	2,751,712	TCTGAAATGTCAAGTGGGCC	TCGACTTGAAGCATCACACC
10-18337	10	18,337,098	862,250	GACCTATCCAGTGACCTTTCGAG	ATGACCTATCCAGTGACCTTTCGAA

Primer name	chr	Physical distance	Distance（bp）	Forward primer	Reverse primer
10-19026	10	19,025,553	688,455	GATGACAGTATTACA GGTAGCTCTGT	ATGACAGTATTACAG GTAGCTCTGC
RM25716	10	19,273,389	247,836	AATCTTCCTCGAGCC AATGATGC	CAGACACAACACAAT TCCATGTGC
chr10-95.6	10	21,533,254	2,259,865	GATTGACAAGAAGGA GGGTGGTT	AGCAGCTTAGCTCGT AGCCCGTAG
chr10-106.0	10	22,919,087	1,385,833	TAATCCAACCAGTTC ATTCAG	TACACCCCACTGCCC TGCTACAC
chr11-2.5	11	2,523,692		CGACCTGACCCAAAT ACCCC	AACAAGGAAGAAGG GTGCCG
chr11-23.1	11	2,844,126	320,434	AGCATCAATTCAGCT TGCTTGC	TTCTGGTTCTTTGAGA GAGTGTGC
11-3494	11	3,494,009	649,883	GCATCGAGTCCTTCC ACAGTTC	AGCATCGAGTCCTTC CACAGTTT
11-4425	11	4,424,877	930,868	CATGGAGAGGTTTGT TGTTACGCAT	CATGGAGAGGTTTGT TGTTACGCAA
11-6480	11	6,480,237	2,055,360	CTGCGGCCAGATTGC TG	AAGCTCTGCGGCCAG ATTGCTA
chr11-37.6	11	8,990,534	2,510,297	TCTCTCCTCTTGTTTG GCTC	ACACACCAACACGAC CACAC
chr11-48.2	11	9,368,443	377,909	TGCTGAATGGTTGTTT CTGGA	AGGCCAACAATCACA TTTCAGA
RM4862	11	9,884,968	516,525	ATCACAGTGTTGATC CATCTCC	CAATCCTAAGAAGGG TATCTGG
11-10742	11	10,742,238	857,270	AGGTGGCAAGTTAGT CAGACAG	AGTAGGTGGCAAGTT AGTCAGACAT
RM6091	11	13,290,144	2,547,906	GCGGACACACCAGA GAATAAGC	GTGCTGTCCTGTCCTT GAATCC
chr11-61.2	11	13,869,045	578,901	GCGGACACACCAGA GAATAAGC	GTGCTGTCCTGTCCTT GAATCC
11-15251	11	15,251,102	1,382,057	TGCCACAGCCACTAC AGTGT	GCCACAGCCACTACA GTGC
RM287	11	16,610,716	1,359,614	GGCTACACCTACACG CGAGAACC	AGATGCATGGAATGC CTGTTTGG
chr11-69.5	11	19,351,669	2,740,953	ATGAGGTCCTATGTTT AAGCAGT	ATTGAAGCATCTTTA GGGTTTGG
11-20777	11	20,777,287	1,425,618	CACCGGAAATACGGT TCATTGACAA	ACCGGAAATACGGTT CATTGACAG
chr11-81.1	11	22,640,888	1,863,601	CGGCTTCGGCTTCTTC TTCC	GACACTCCCGTTTGT ATAACTGTTGC
chr11-90.3	11	23,972,667	1,331,779	CCACGTGTCAGTCAT CCATCTAGG	GGTCTGCTCGATTAC CATCAAACTCC

Primer name	chr	Physical distance	Distance（bp）	Forward primer	Reverse primer
chr12-0.1	12	2,510		TGTAGCTAGGGTTCG CTGGT	CGGAGTGGAGTTGCT TTCTC
chr12-21.6	12	2,182,902	2,180,392	GAGTCACGGGATGTT GCC	CACAAAGGCGTGTGG GTTAG
RM27722	12	5,464,928	3,282,026	GATGCAACCACAACC ACTTAGTAACC	TTCGCTTACTGATCGT CCTACAAAGC
12-5567	12	5,567,344	102,416	GCCGAGCCGAAGTCT GCAAC	CGCCGAGCCGAAGTC TGCAAT
chr12-43.1	12	8,025,081	2,457,737	GTCATCGTGAGGCCC ATTAAGC	GGAAGAAGAAGACG TCCTGTAGCC
chr12-54.9	12	8,829,295	804,214	AAGTAGTGGTCGAAG TGTGTATCG	GGTGAATGGTCAAGT GACTTAGG
12-10541	12	10,541,144	1,711,849	AATTGTTCTATTTGCG TGGAAAGTGAC	CAATTGTTCTATTTGC GTGGAAAGTGAT
12-12388	12	12,388,405	1,847,261	TACCACCTGTTGCTA GAAGCCA	ACCACCTGTTGCTAG AAGCCG
RM1261	12	17,578,154	5,189,749	ATGGTAGAGACACAA GTCCATGC	GACAAATTGGTGTAG GTGAAGG
chr12-72.4	12	17,982,442	404,288	TGCACATTACATCCTC AGATCACC	TCTTCAGTTTCTTCCT CCTCTCTGC
chr12-84.4	12	18,310,512	328,070	CATTCTACCGATGATT GCAGAGG	CTACATTAAGCGTGA GCGACAGC
RM28367	12	21,173,248	2,862,736	CGTATCTCCACCTCCC GAGAAGC	GCCAAATCTCACGGA TCGAAGC
chr12-96.4	12	24,620,830	3,447,582	TGGCCTGTAATCTAG CACACTTCC	GGCTTCATCTTTGCCA CAGC
chr12-128.9	12	27,164,987	2,544,157	GTATTGGTCACCGGT CTAAGTCG	ATTATTGGCCCTAGGT CAACACC
chr12-154.8	12	27,465,825	300,838	CCCTTCTCTTCTCCTG TACCC	GGTTCCTAGGTGTCAT CTTAGTGC

Table S4. Summary of RNA sequencing and alignment data for each sample

**Sample**[a]	**Raw Reads**	**Clean Reads**	**Clean Base(G)**	**Error Rate(%)**	**Q20(%)**	**Q30(%)**	**GC Content(%)**
DW14ck_3h_1	50091174	49185402	7.38	0.01	99.1	96.4	50.9
DW14ck_3h_2	47200534	46459374	6.97	0.01	99.1	96.5	50.6
DW14ck_3h_3	47201498	46394214	6.96	0.01	99.2	96.8	50.7
DW14S_3h_1	46894294	46079604	6.91	0.01	99.0	96.4	50.1
DW14S_3h_2	46170160	45498810	6.82	0.01	99.2	96.7	50.1
DW14S_3h_3	48950548	48430658	7.26	0.01	99.2	96.6	49.7
DW14ck_12h_1	49557968	48449722	7.27	0.01	99.2	96.5	48.8
DW14ck_12h_2	46250992	45531538	6.83	0.01	99.2	96.7	49.1
DW14ck_12h_3	49325348	48546202	7.28	0.01	99.2	96.7	47.1
DW14S_12h_1	47233946	46469376	6.97	0.01	99.1	96.4	48.8
DW14S_12h_2	50373404	49530066	7.43	0.01	99.2	96.6	48.7
DW14S_12h_3	49654294	48777212	7.32	0.01	99.2	96.8	49.6
DW14ck_24h_1	47831032	47341966	7.1	0.01	99.2	96.8	49.8
DW14ck_24h_2	50728346	49854030	7.48	0.01	99.3	96.9	50.0
DW14ck_24h_3	47688088	46786564	7.02	0.01	99.3	96.9	49.6
DW14S_24h_1	54399780	53674924	8.05	0.01	99.3	97.3	49.5
DW14S_24h_2	46739824	46055538	6.91	0.01	99.3	97.2	50.2
DW14S_24h_3	52158604	51425818	7.71	0.01	99.3	97.3	49.8
DW14ck_48h_1	47235890	46352962	6.95	0.01	99.3	97.1	50.7
DW14ck_48h_2	50168632	49299698	7.39	0.01	99.3	97.1	50.4
DW14ck_48h_3	47737616	46952258	7.04	0.01	99.3	97.3	50.5
DW14S_48h_1	49329962	48414222	7.26	0.01	99.3	97.2	50.4
DW14S_48h_2	48530234	47417766	7.11	0.01	99.3	97.2	49.5
DW14S_48h_3	45197562	44362050	6.65	0.01	99.3	97.2	50.6

[a] DW14S_3h_1 is the first biological replicate of DW14 after 3 hours of saline treatment; DW14CK_3h_1 is the control group's first replicate at the same time point.

Table S5. The FPKM value of deferentially expressed genes in *qSTS5* region.

gene_id	DW14ck_3h<br>Mean±SD	DW14S_3h<br>Mean±SD	DW14ck_12h<br>Mean±SD	DW14S_12h<br>Mean±SD	DW14CK_24h<br>Mean±SD	DW14S_24h<br>Mean±SD	DW14CK_48h<br>Mean±SD	DW14S_48h<br>Mean±SD
Os05g0331900	0.4±0.2	0.1±0.1	3.3±0.5	0.3±0.1	0.6±0.2	0.2±0.0	0.5±0.2	0.2±0.1
Os05g0341450	0.6±0.2	0.2±0.1	1.7±0.3	0.1±0.1	0.6±0.1	0.2±0.0	0.2±0.1	0.1±0.0
Os05g0349800	1.0±0.2	19.0±2.5	0.4±0.1	3.8±0.7	0.5±0.1	7.4±0.7	0.1±0.0	4.4±0.8
Os05g0361700	3.8±0.6	36.1±4.4	1.4±0.4	3.9±0.5	2.9±0.4	13.1±2.1	2.7±0.4	8.3±0.7
Os05g0373900	5.9±0.6	20.0±0.5	4.9±0.2	23.8±1.5	2.2±0.6	30.1±1.4	2.7±0.1	19.8±1.4
Os05g0381400	4.5±0.9	141.1±3.2	0.6±0.4	7.6±1.1	2.0±0.9	45.0±3.8	0.6±0.2	19.4±1.4
Os05g0390300	1.5±0.2	0.5±0.1	0.2±0.1	1.0±0.1	2.9±0.5	0.7±0.1	2.6±0.4	0.2±0.1
Os05g0399400	22.5±0.5	223.5±10.0	37.7±3.6	388.6±12.0	11.7±1.0	190.4±13.5	19.7±2.0	120.7±2.3
